# Very long-chain fatty acids are accumulated in triacylglycerol and nonesterified forms in colorectal cancer tissues

**DOI:** 10.1038/s41598-021-85603-w

**Published:** 2021-03-17

**Authors:** Kotaro Hama, Yuko Fujiwara, Tamuro Hayama, Tsuyoshi Ozawa, Keijiro Nozawa, Keiji Matsuda, Yojiro Hashiguchi, Kazuaki Yokoyama

**Affiliations:** 1 Faculty of Pharma-Sciences, Teikyo University, Teikyo University School of Medicine, 2-11-1 Kaga, Itabashi-ku, Tokyo, 173-8605 Japan; 2 Department of Surgery, Teikyo University School of Medicine, 2-11-1 Kaga, Itabashi-ku, Tokyo, 173-8605 Japan

**Keywords:** Tumour biomarkers, Fatty acids, Metabolomics, Colorectal cancer

## Abstract

Colorectal cancer (CRC) is a major cancer, and its precise diagnosis is especially important for the development of effective therapeutics. In a series of metabolome analyses, the levels of very long chain fatty acids (VLCFA) were shown to be elevated in CRC tissues, although the endogenous form of VLCFA has not been fully elucidated. In this study we analyzed the amount of nonesterified fatty acids, acyl-CoA species, phospholipids and neutral lipids such as cholesterylesters using liquid-chromatography–mass spectrometry. Here we showed that VLCFA were accumulated in triacylglycerol (TAG) and nonesterified forms in CRC tissues. The levels of TAG species harboring a VLCFA moiety (VLCFA-TAG) were significantly correlated with that of nonesterified VLCFA. We also showed that the expression level of elongation of very long-chain fatty acids protein 1 (*ELOVL1*) is increased in CRC tissues, and the inhibition of *ELOVL1* decreased the levels of VLCFA-TAG and nonesterified VLCFA in CRC cell lines. Our results suggest that the upregulation of *ELOVL1* contributes to the accumulation of VLCFA-TAG and nonesterified VLCFA in CRC tissues.

## Introduction

Globally, colorectal cancer (CRC) is the second and third highest cause of cancer in women and men, respectively, and is also the second most common cause of cancer-related death in the United States and Japan^1^. A number of lifestyle factors have been implicated as risk factors for CRC. More specifically, a westernized diet characterized by a higher intake in total energy, saturated fat, sucrose and salt, and a lower intake in fiber, has often been described as the major risk factor for CRC. A recent cohort study reported that the intake of *n*-3 polyunsaturated fatty acids (PUFA) reduced the risk of CRC occurrence, whereas the ratio of *n*-3 PUFA/*n*-6 PUFA was not correlated with that^2^, These results suggested that the pathological contribution of fatty acids and lipid metabolism in CRC remains to be elucidated.

Very long-chain fatty acids (VLCFA) with no less than 23 or 24 carbons are known to be endogenously synthesized as very long-chain fatty acyl-CoA (VLCFA-CoA) through a fatty acid elongation process^3^. Then, VLCFA-CoA are incorporated into complex lipids, such as phospholipids (PL). Recently, analysis using gas chromatography-mass spectrometry (GC–MS) revealed that the levels of VLCFA were elevated in plasma and surgical specimens from patients with CRC^4^. However, the endogenous form of VLCFA in CRC tissues has not been fully understood due to the limited number of intensive lipidomic analyses focusing on VLCFA. Notably, fatty acyl moieties are liberated from complex lipids during the derivatizing process of sample preparation for GC–MS, thus leading to loss of information about the VLCFA-containing endogenous lipid species.

Advances in liquid chromatography-electrospray ionization-mass spectrometry (LC–ESI–MS) have enabled the simultaneous quantification of a large number of underivatized lipid species in biological samples. In this study, we analyzed underivatized lipid fractions in surgical specimens from patients with CRC using LC–ESI–MS, and revealed that VLCFA were accumulated in triacylglycerol (TAG) and nonesterified forms in CRC tissues. To gain a mechanistic insight, we also conducted an expression analysis of gene involved in lipid metabolism in CRC tissues.

## Results

### Levels of non-esterified very long-chain fatty acids were elevated in colorectal cancer tissues

**Figure 1:**
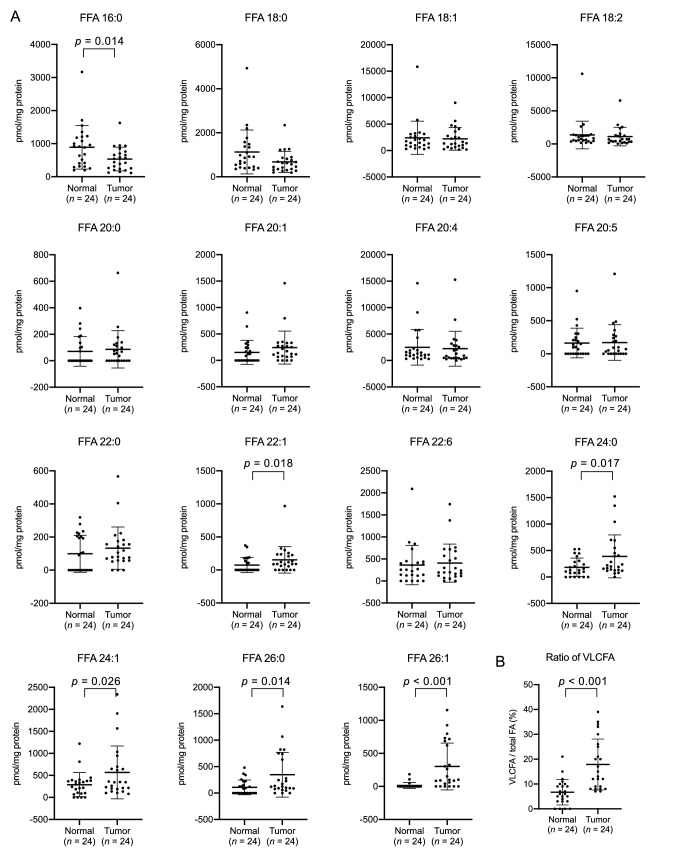
Quantities of nonesterified fatty acid (free fatty acid, FFA) species in the colorectal cancer (CRC) tissues. Each FFA species (**A**) and the portion of nonesterified VLCFA (**B**) was analyzed using LC–MS. All of the nonesterified VLCFA comprising FFA 24:0, FFA 24:1, FFA 26:0 and FFA 26:1 were accumulated in CRC tissues. Data represent the mean ± SD. Statistical analysis was performed using the two-tailed paired *t*-test in (A and B).

We first profiled the nonesterified fatty acid (free fatty acid, FFA) species in CRC tissues using the LC–MS method. To analyze the underivatized FFA in total lipid fractions, we used a C_8_ reversed-phase column and mobile phases adjusted to pH 9.0 by ammonium hydroxide, which are almost identical with the current methods used for the analysis of acyl-CoA^5^. We validated our method using two isotopically labeled palmitic acids, and confirmed that 8–2000 pmol/injection of FFA species could be quantified with the present quantitative method (Supplementary Table S1 online). We then profiled each FFA species in CRC tissues from 24 patients. We accordingly observed that the amount of FFA 16:0 in CRC was significantly lower than that in adjacent normal tissues. By contrast, the amount of nonesterified VLCFA species (FFA 24:0, FFA 24:1, FFA 26:0, and FFA 26:1) and FFA 22:1 was demonstrated to be significantly higher in CRC than in normal tissues (Fig. 1A). We further found that the portion of 4 nonesterified VLCFA species with no less than 24 carbons (FFA 24:0, FFA 24:1, FFA 26:0, FFA 26:1) was also significantly higher in CRC than in adjacent normal tissues (Fig. 1B). These results showed that VLCFA species were accumulated in nonesterified form in CRC tissues.

### Levels of very long-chain fatty acyl-CoA were not elevated in colorectal cancer tissues

**Figure 2:**
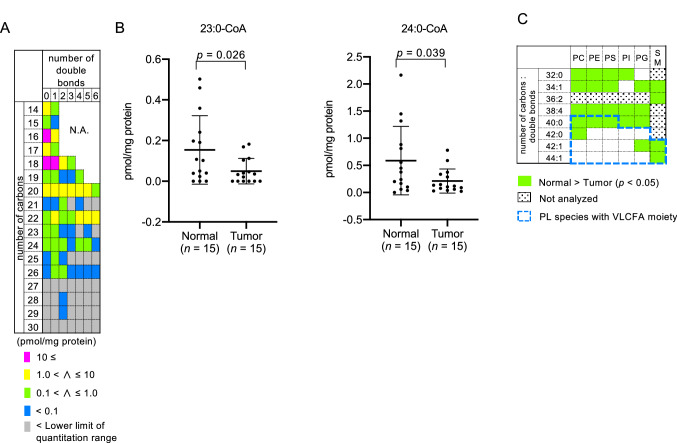
The amount of acyl-CoA, phospholipid (PL) and sphingomyelin (SM) species in colorectal cancer (CRC) tissues. (**A**) Each acyl-CoA species in adjunct normal tissues (*n* = 15) was quantified and classified according to the number of carbon and double bonds in its acyl moiety. The ratio of the peak area for each acyl-CoA/D_31_-16:0-CoA was used to calculate the amount of each acyl-CoA species. The mean quantity of each acyl-CoA species in the tissues is represented with a color key. Data are also summarized in Supplementary Table S2 online. Acyl-CoA species observed to be present below the quantitation range are indicated in gray. The total amount of the top three acyl-CoA species (16:0-CoA, 18:0-CoA and 18:1-CoA) comprised more than 50% of the total amount of all acyl-CoA species, analyzed within the quantitative range. (**B**) The amount of 23:0-CoA and 24:0-CoA species in CRC and adjacent normal tissues. The amount of 23:0-CoA and 24:0-CoA species in CRC tissues was significantly lower than in adjunct normal tissues. (**C**) The amount of each PL and SM species was quantified using LC–MS in the positive ion mode. Lipid species present in quantities significantly lower in CRC (*n* = 24) relative to adjacent normal tissues (*n* = 24) are presented in lime. PL and SM species for which the multiple reaction monitoring (MRM) channels were not designed are indicated in the dot box. PL and SM species with a VLCFA moiety (C24:0 and C26:0) are indicated by the blue dashed line. Data represent the mean ± SD. Statistical analysis was performed using the two-tailed paired *t*-test in (**B**,**C**).

As is known, VLCFA are initially produced as VLCFA-CoA through a de novo fatty acid elongation process^3^. Therefore, we next profiled the amount of VLCFA-CoA species in CRC tissues. Among 59 acyl-CoA species quantified in CRC and adjacent normal tissues, we found that 3% of the total acyl-CoA species consisted of VLCFA-CoA species, with no less than 23 carbons in their fatty acyl moiety. We noticed that tetracosenoyl-CoA (24:1-CoA) was the most abundant VLCFA-CoA species in both CRC and adjacent normal tissues (Fig. 2A, and Supplementary Table S2 on line). However, we could not observe a significant accumulation of any VLCFA-CoA species in CRC tissues, but rather found that the amount of 2 VLCFA-CoA species (23:0-CoA and 24:0-CoA) was significantly lower in CRC than normal tissues (Fig. 2B). These results showed that the intracellular pool of VLCFA-CoA was not significantly increased in CRC tissues.

### Levels of phospholipid (PL) and sphingomyelin (SM) species containing a very long-chain fatty acyl moiety were not altered in colorectal cancer tissues

Both PL and SM are known to be abundantly present as membrane components in cells, and thus serving as an intracellular reservoir for free fatty acids. We next analyzed the PL and SM species to examine whether VLCFA species were accumulated in esterified forms in CRC tissues. We analyzed 39 PL species (7 phosphatidylcholine (PC), 7 phosphatidylethanolamine (PE), 7 phosphatidylserine (PS), 7 phosphatidylinositol (PI), 7 phosphatidylglycerol (PG) and 4 SM species), in which the acyl moieties were determined by scanning the product ions from parental ions of each PL species. We accordingly observed that the amount of most PL and SM species (17 out of 19 species) with long-chain fatty acyl moieties (C16:0, C18:0, C18:1, C20:4 and C22:0) and lacking a VLCFA moiety was significantly lower in CRC compared with adjacent normal tissues, whereas the amount of only 7 out of 20 PL and SM species with a VLCFA moiety (C24:0 and C26:0) was lower in CRC tissues (Fig. 2C and Supplementary Fig. S1 online). We could not observe any PL or SM species with a significantly higher amount in CRC tissues. These results showed that VLCFA were not accumulated as esterified forms in PL or SM species.

### Levels of triacylglycerol species containing a very long-chain fatty acyl moiety were elevated in colorectal cancer tissues

**Figure 3:**
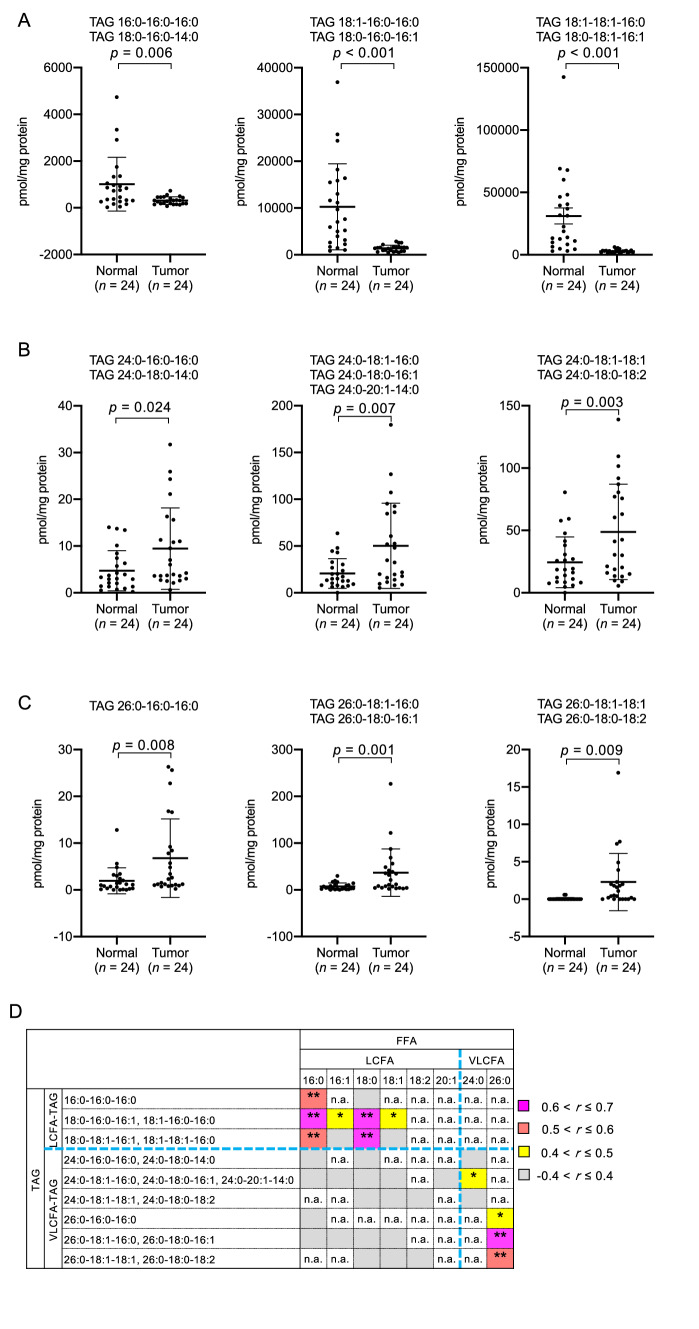
Quantities of each triacylglycerol (TAG) species in colorectal cancer (CRC) tissues. Each TAG species was analyzed using LC-MS. (**A**) The amount of 6 TAG species containing long chain fatty acyl moieties (C14–C18) without VLCFA was significantly lower in CRC tissues. (**B**,**C**) All 7 and 5 TAG species containing C24:0 (B) and C26:0 (**C**), respectively, were more abundant in CRC tissues. (**D**) The correlation between the difference (CRC – normal) of TAG and FFA species. The correlation between each TAG and FFA species present in each TAG species was represented according to the value of the correlation coefficient. The amount of TAG species containing VLCFA was positively correlated with the amount of nonesterified very long-chain fatty acids. The correlation that was not analyzed due to the absence of FFA in the corresponding TAG species is represented as "n.a." The 3 acyl-moieties for each TAG species were determined by the product ion spectra obtained through PRM analysis, and are represented in the left column in the matrix. Fragment ion spectra for each TAG species are listed in the Supplementary Figure S5 online. Data represent the mean ± SD. Statistical analysis was performed using the two-tailed paired *t*-test in (**A**–**C**) and the Pearson correlation coefficient in (**D**). *p < 0.05, **p < 0.01.

Nonesterified VLCFA is known to be produced from neutral lipids such as TAG and cholesterylesters (CE) by lipases. Therefore, we profiled each TAG and CE species in CRC tissues. We found that the amount of TAG species containing long-chain fatty acyl moieties (C14:0, C16:0, C16:1, C18:0 and C18:1) and lacking a VLCFA moiety was significantly lower in CRC than in adjacent normal tissues (Fig. 3A). In contrast, the amount of TAG species with one VLCFA moiety (C24:0 or C26:0) and two long-chain fatty acyl moieties (VLCFA-TAG) was significantly higher in CRC tissues (Fig. 3B,C). We further observed that the levels of each TAG species were significantly correlated with those of FFA species present in each TAG species (Fig. 3D, Supplementary Fig. S2 online). However, we could not observe any significant differences in the CE species between CRC and normal tissues (Supplementary Fig. S3 online). These results showed that VLCFA were accumulated as esterified forms in TAG species, indicating that the TAG metabolism might be involved in the accumulation of nonesterified VLCFA in CRC tissues.

### Levels of expression of ELOVL1 and ELOVL5 was increased in colorectal cancer tissues

**Figure 4:**
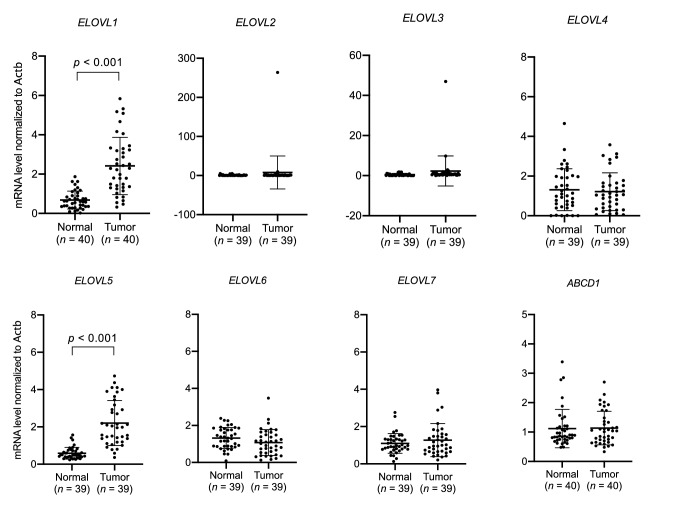
*ELOVL1* and *ELOVL5* were highly expressed in colorectal cancer (CRC) tissues. The mRNA level of *ELOVL1*–*ELOVL7* and *ABCD1* in CRC and adjacent normal tissues. Data represent the mean ± SD. Statistical analysis was performed using the two-tailed paired *t*-test.

To explore the machinery underlying the accumulation of VLCFA in CRC tissues, we first focused on the process of fatty acyl elongation catalyzed by 7 elongation of very long-chain fatty acids proteins (ELOVL1–ELOVL7). We examined the mRNA levels of *ELOVL1*–*ELOVL7* in samples from 40 patients with CRC, and found that the expression levels of *ELOVL1* and *ELOVL5* were significantly higher in CRC than in adjacent normal tissues (Fig. 4), consistent with a previous report^6^. We also found that the expression level of ATP-binding cassette transporter subfamily D1 (*ABCD1*), which is known to be critical for the degradation of VLCFA^7^, was not significantly altered (Fig. 4). In particular, ELOVL1 has been shown to play critical roles in the de novo synthesis of VLCFA^3^. In addition, ELOVL1 and ELOVL5 have been demonstrated to prefer VLCFA-CoA (C22–C26) and polyunsaturated fatty acyl-CoA (18:2-CoA and 18:3-CoA) as substrates, respectively^8^. Considering the substrate specificities of ELOVL, our results suggested that ELOVL1 was involved in the accumulation of VLCFA in CRC tissues.

### ELOVL1 contributed to the accumulation of nonesterified and esterified very long-chain fatty acids in cultured cells

**Figure 5:**
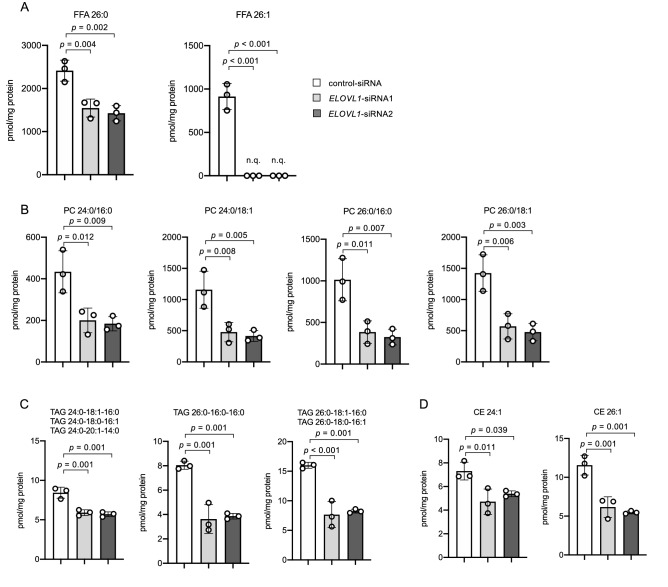
Quantities of FFA, PC, TAG and CE species in HCT116 colorectal cancer cells transfected with *ELOVL1* siRNA. (**A**) The amount of nonesterified VLCFA significantly altered by siRNA treatment. "n.q.", not quantified due to the signal being smaller than the lower limit of the quantitation range. (**B**) The amount of each PC species with a VLCFA moiety. The acyl-moieties for each PC species were determined by LC–MS/MS/MS in the negative ion mode. (**C**,**D**) The amount of each VLCFA-TAG (**C**) and VLCFA-CE (**D**) species that was significantly altered by siRNA treatment. The acyl-moieties for each TAG and CE species were determined by the product ion spectra obtained through PRM analysis. The amount of both the nonesterified (FFA) and esterified VLCFA (PC, TAG and CE) was significantly decreased in HCT116 cells transfected with *ELOVL1* siRNA. Data represent the mean ± SD. Statistical analysis was performed using the Dunnett’s *t*-test in (**A**–**D**).

We finally confirmed the role of ELOVL1 in the accumulation of VLCFA. We noticed that the amount of nonesterified VLCFA species (FFA 26:0 and FFA 26:1) and VLCFA-TAG species harboring C24:0 or C26:0 as their acyl moiety was significantly decreased in HCT116 colorectal cancer cells transfected with *ELOVL1* siRNA (Fig. 5A,C). In addition, treatment with *ELOVL1* siRNA was also shown to lead to a decrease in the amount of PC and CE species harboring C24:0, C24:1, C26:0 or C26:1 as their acyl moiety (Fig. 5B,D). By contrast, we found that the amount of nonesterified VLCFA species (FFA 24:0, FFA 24:1, FFA 26:0 and FFA 26:1) and VLCFA-TAG species harboring C24:0 or C26:0 as their acyl moiety was significantly increased in HEK293T cells exogenously overexpressing ELOVL1 (Supplementary Fig. S4A,E online). In addition, we observed that overexpression of ELOVL1 also increased the amount of VLCFA-CoA (24:0-CoA, 24:1-CoA, 26:1-CoA and 28:1-CoA), VLCFA-PL (3 PC, 1 PE, 4 PS and 2 PI species) and VLCFA-CE species (harboring C24:0, C24:1, C26:0 or C26:1) as their acyl moiety (Supplementary Fig. S4B–D,F and Supplementary Table S3 online). These results suggested that ELOVL1 contributed to the levels of both nonesterified and esterified VLCFA in CRC cells.

## Discussion

Our lipidomic study revealed that VLCFA were accumulated in TAG and nonesterified forms in CRC tissues (Figs. 1, 3). These results were partially consistent with a previous report, in which FFA 24:0 and FFA 24:1 were shown to be accumulated in both CRC tissues with and without lymph node metastasis^9^. However, it will be necessary to examine whether esterified VLCFA is accumulated in other lipid species such as glycolipids in CRC tissues. It is also interesting to identify the microscopic localization of VLCFA using Imaging-MS system to clarify which cells in mucosa (or submucosa, in case) are responsible for the accumulation of VLCFA in CRC tissues. Note that TAG species with long chain acyl moieties, such as TAG 18:1–18:1–16:0, which accounted for the majority in the total TAG fraction, were significantly decreased in CRC tissues (Fig. 3A), supporting the previous result that the level of TAG was lower in CRC compared with normal tissues^10^. It has also been shown that the amount of total VLCFA, including esterified and nonesterified forms, was elevated in the sera of patients with CRC^11^. However, whether the accumulation of VLCFA in CRC tissues directly leads to changes in the composition of serum fatty acids would require further examination through the comparison of the composition of fatty acids in the sera of pre- and post-surgery patients.

As the levels of TAG and nonesterified FA species were significantly correlated (Fig. 3B and Supplementary Fig. S2 online), we assumued that nonesterified VLCFA might be liberated from VLCFA-TAG species by lipases. The suppression or overexpression of ELOVL1 altered the levels of all analyzed esterified VLCFA species including TAG, CE, PL, SM and acyl-CoA in cultured cells (Fig. 5 and Supplementary Fig. S4 online), whereas VLCFA were accumulated only in TAG as esterified forms in CRC tissues (Fig. 3). This discrepancy might be explained by other enzymes involved in the de novo TAG synthesis. For example, phospholipase C is known to degrade PC into diacylglycerol, which is a precursor for TAG synthesis. Upregulation of the activity of phospholipase C in CRC tissues would lead to the efficient degradation of VLCFA-PC, resulting in the accumulation of VLCFA-TAG.

Further studies are required to address the pathophysiological functions of VLCFA in CRC. Recently it was shown that treatment with hexacosanoic acid (C26:0) induced the endoplasmic reticulum stress in fibroblasts from patients with X-linked adrenoleukodystrophy^12^. Since excessive endoplasmic reticulum stress is involved in tumorigenesis^13^, it might be possible that the accumulation of nonesterified VLCFA or VLCFA-TAG is involved in the CRC development through the abnormal activation of endoplasmic reticulum stress.

## Materials and methods

### Ethics

This study abided by the Declarations of Helsinki Principles, and the research protocol was approved by the Ethics Committee of Teikyo University (#19-153). We enrolled 40 patients treated with curative resection at the Teikyo University Hospital, Japan during the 2019–2020 period. Surgery was elective for all patients. Informed consent was obtained from all participants and the reporting of our research is in accordance with the STROBE guidelines^14^.

### Reagents

PC 15:0/D_7_-18:1, PE 15:0/D_7_-18:1, PS 15:0/D_7_-18:1, PI 15:0/D_7_-18:1, PG 15:0/D_7_-18:1 and SM d18:1/D_9_-18:1 were purchased from Avanti Polar Lipids, Inc. (Alabaster, AL, USA). All chemicals used in the mobile phases were purchased from Fujifilm Wako Pure Chemical Corporation (Osaka, Japan). Deuterium-labeled (7, 7, 8, 8-D_4_) palmitic acid (FA D_4_-16:0) and FA D_31_-16:0 were purchased from Cambridge Isotope Laboratories, Inc. (Andover, MA, USA), and Santa Cruz Biotechnology (Dallas, TX, USA), respectively. Two *ELOVL1* siRNAs and a control siRNA were obtained from *Silencer* select RNAi (Thermo Fisher Scientific, Waltham, MA, USA) and MISSION siRNA Universal Negative control (Sigma-Aldrich, Inc., St. Louis, MO, USA), respectively.

### Sample preparation

All colorectal tissue specimen were dissected into small pieces, approximately 5 mm a side, frozen in liquid nitrogen within 24 h after surgery, and stored at − 80 °C until lipid extraction. Normal tissues were obtained by lifting and dissecting the mucosa layer. The specimens mainly consisted of mucosa tissues but did not contain circular or longitudinal muscles. Due to the limited size of each tissue specimen, we prepared samples for FFA, PL, TAG and CE analysis from 24 of the 40 patients enrolled in this study. Sample preparation for FFA, PL, TAG and CE analysis was conducted as previously reported with slight modification^15^. Briefly, tissue specimens were homogenized in 1 mL methanol using a Micro Smash MS-100R instrument (TOMY, Tokyo, Japan) at 3000 rpm for 10 s at 4 °C. This procedure was repeated 3 to 6 times until tissues were fully homogenized. Then, an internal standard (IS) mixture composed of 0.05 nmol of PC 15:0/D_7_-18:1, PE 15:0/D_7_-18:1, PS 15:0/D_7_-18:1, PI 15:0/D_7_-18:1, PG 15:0/D_7_-18:1, SM d18:1/D_9_-18:1, and 2 nmol FA D_4_-16:0 was added to the tissue homogenate, and the total lipid fraction was extracted by the Bligh and Dyer method^16^. The resulting lower organic phase was evaporated using an EZ-2 centrifugal evaporator (Genevac, Ipswich, UK), and the resulting precipitate was reconstituted with 0.1 mL ethanol, followed by filtration with a YMC Duo-Filter (4 mm i.d., pore size 0.2 µm; YMC Co., Ltd., Kyoto, Japan). Samples were stored at − 20 °C until further analysis. For the acyl-CoA analysis, we prepared samples from 15 of the 40 patients enrolled in this study. Tissue specimens were homogenized in 0.9 mL of acetonitrile/isopropanol (3:1 by volume) and the acyl-CoA fraction was extracted as previously described^5^. Samples were stored at − 20 °C until further analysis. Homogenate protein concentrations were determined using a BCA protein assay kit (Thermo Fisher Scientific). The samples used for the validation of the LC–MS method for FFA were prepared as described in Supplemental Materials online.

### LC–MS/MS analysis

Quantitation of each PL species was conducted using a TSQ Quantum Ultra (Thermo Fisher Scientific) linked to an Accela HPLC system (Thermo Fisher Scientific). An InertSustain C_18_ metal-free column (2.1 mm i.d. × 50 mm, particle size 3.0 µm; GL Sciences, Tokyo, Japan) was used at 50 °C. Mobile phases were acetonitrile/methanol/water (2:2:1, by vol.) with 0.1% formic acid and 0.028% ammonia (A), and isopropanol with 0.1% formic acid and 0.028% ammonia (B). The programmed solvent gradient consisted of solvents A/B at a 70:30 ratio for 1 min, linear alteration to 5:95 over 10 min, hold at 5:95 over 4 min, linear conversion to 70:30 over 1 min and then hold at 70:30 over 2.5 min. The flow rate was 280 μL/min, and the volume of injected samples was 5 μL. Analysis was conducted in the positive ion mode. The period for data collection was 25 ms/cycle for each multiple reaction monitoring (MRM) transition. The following conditions were used for positive ion MRM: ion spray voltage, 3.5 kV; vaporizer temperature, 287 °C; sheath gas pressure, 10 psi; auxiliary gas pressure, 35 psi; collision gas pressure, 0.7 mTorr; capillary temperature, 320 °C; tube lens offset 133 V. Nitrogen was used as sheath and auxiliary gas, whereas argon was used as collision gas. The Xcalibur software (Thermo Fisher Scientific) was used for data acquisition and processing. The fatty acyl moieties in each PL species were determined through structural analysis in the negative ion mode conducted with a QTRAP 4500 (Sciex, Framingham, MA, USA) linked to a Nexera XR HPLC system (Shimadzu Corp., Kyoto, Japan) as previously described^15^. The fatty acyl moieties of each PL were assigned through the detection of the product ions from fatty acyl residues and lysophospholipids. In addition, fatty acyl residue positions were also determined by comparing the spectral intensity of two lysophospholipid ions produced from precursor ions, as previously reported^17^. Quantitation of each FFA species was conducted using a TSQ Quantum Ultra linked to an Accela HPLC system. A Capcell Pak C_8_ UG120 column (1.5 mm i.d. × 35 mm, particle size 5.0 µm; OSAKA SODA, Co., Ltd., Osaka, Japan) was used at 40 °C. Mobile phases were 5 mM ammonium formate in water (pH 9.0) (A) and 5 mM ammonium formate in a water/isopropanol solution (5:95 by volume; pH 9.0) (B). The programmed solvent gradient consisted of solvents A/B at a 70:30 ratio for 1 min, linear alteration to 5:95 over 10 min, hold at 5:95 over 4 min, linear conversion to 70:30 over 1 min and then hold at 70:30 over 2.5 min. The flow rate was 200 μL/min, and the volume of the injected samples was 10 μL. Analysis was conducted in the negative ion mode. The period for data collection was 40 ms/cycle for each MRM transition. The following conditions were used for negative ion MRM: ion spray voltage, 2.5 kV; vaporizer temperature, 0 °C; sheath gas pressure, 10 psi; auxiliary gas pressure, 35 psi; collision gas pressure, 0.7 mTorr; capillary temperature, 270 °C; tube lens offset 146 V. Nitrogen was used as sheath and auxiliary gas, whereas argon was used as collision gas. Each MRM transition for PL and FFA species used in the LC–MS/MS analysis is listed in Supplementary Table S4 online. The Xcalibur software was used for data acquisition and processing. Quantitation of each acyl-CoA species was conducted using a QTRAP 4500 linked to a Nexera XR HPLC system as described previously^5^. Quantitation of each TAG and CE species was conducted using a Q-Exactive Orbitrap MS (Thermo Fisher Scientific) linked to an Ultimate 3000 ultra high performance LC system (Thermo Fisher Scientific). A CAPCELL PAK C_18_ ACR column (1.5 mm i.d. × 100 mm, particle size 3.0 µm; OSAKA SODA, Co., Ltd.) was used at 50 °C. Mobile phases were acetonitrile/methanol/water (2:2:1, by vol.) with 0.1% formic acid and 0.028% ammonia (A), and isopropanol with 0.1% formic acid and 0.028% ammonia (B). The programmed solvent gradient consisted of solvents A/B at a 100/0 ratio for 5 min, linear alteration to 80/20 over 4 min, linear alteration to 20/80 over 66 min, hold at 20/80 over 5 min, linear conversion to 100/0 over 5 min and then hold at 100/0 over 4 min. The flow rate was 280 μL/min, and the volume of the injected samples was 2 μL. Analysis was conducted in the positive ion mode using the parallel reaction monitoring (PRM) method. The following conditions were used for positive ion PRM: resolution, 35,000; stepped nce, 30; AGC target, 2e^5^; Maximum IT, 100 ms; Isolation window, 1.0 m*/z*; ion spray voltage, 1.5 kV; capillary temperature, 250 °C; S-lens RF level 50 V. Nitrogen was used as sheath, auxiliary and collision gas. The Xcalibur software was used for data acquisition and processing. PC 15:0/D_7_-18:1 was used for the tentative IS for TAG and CE. The fatty acyl moieties in each TAG and CE species were determined according to the product ion spectra obtained through the PRM analysis. The precursor and product ions to obtain the chromatogram to quantify each TAG and CE species and PC 15:0/D_7_-18:1 are listed in Supplementary Table S5 online.

### Quantitative real-time RT-PCR

Total RNA from colorectal tissues was extracted using the ISOGEN kit (Nippongene, Toyama, Japan) and cDNA libraries were synthesized using a high capacity cDNA RT kit (Thermo Fisher Scientific). We prepared cDNA samples from 40 patients enrolled in this study, and used them for the analysis of *ELOVL1* and *ABCD1*. Due to shortage of a sample, 39 among 40 cDNA samples were used for the *ELOVL2*–*ELOVL7* analysis. The sequences of the oligonucleotides used in the *ELOVL1*–*ELOVL7* PCR reaction were obtained from PrimerBank (https://pga.mgh.harvard.edu/primerbank/) and are listed in Supplementary Table S6 online.

### Suppression or overexpression of ELOVL1 in cultured cells

HCT116 colorectal cancer cells and HEK293T cells were obtained from Riken Cell Bank (Riken Bioresource Center, Ibaraki, Japan). HCT116 were cultured in DMEM (low glucose, Thermo Fisher Scientific Inc.) supplemented with 10% fetal bovine serum, 2 mM *L*-glutamine (Thermo Fisher Scientific Inc.), 100 U mL^−1^ penicillin, 100 µg mL^−1^ streptomycin (Sigma-Aldrich, Inc.). HEK293T were cultured in DMEM (Sigma-Aldrich, Inc.) supplemented with 10% fetal bovine serum, 2 mM *L*-glutamine (Thermo Fisher Scientific Inc.), 100 U mL^−1^ penicillin, 100 µg mL^−1^ streptomycin (Sigma-Aldrich, Inc.) and 1% non-essential amino acids (Fujifilm Wako Pure Chemical Corporation). Cells were transiently transfected with the *ELOVL1*-siRNA or pcDNA3.1-human *ELOVL1* plasmid using the Lipofectamine 2000 reagent (Thermo Fisher Scientific Inc.), according to the manufacturer’s instructions. Then 2 (*ELOVL1*-siRNA) or 3 (pcDNA3.1-human *ELOVL1* plasmid) days after transfection, cell layers were washed with phosphate buffered saline, scraped from dishes, homogenized in methanol, and used for sample preparation for LC–MS analysis as previously described^5^. Homogenate protein concentrations were determined using a BCA protein assay kit (Thermo Fisher Scientific Inc.).

The sequence of siRNA used were 5′-GCUUCAUGAUUGUCUACAA-3′ (*ELOVL1*-siRNA1) and 5′-UGAUCUUUAUUCUCCGAAA-3′ (*ELOVL1*-siRNA2). The pcDNA3.1-human *ELOVL1* plasmid was constructed as previously described^18^.

### Statistical methods

Statistical analysis was performed using either the Student’s two-tailed *t*-test, two-tailed paired *t*-test, Dunnett’s *t-*test or Pearson’s correlation coefficient. Differences were considered significant if the *p*-value was < 0.05. All statistical analyses were conducted using the IBM SPSS Statistics version 27 (IBM, Armonk, NY, USA).

## Supplementary information

Supplementary information.

## Data Availability

The datasets obtained in the current study are available from the corresponding author on reasonable request.

## Abbreviations

ABCD1: ATP-binding cassette transporter subfamily D1
ACSL: Acyl-CoA synthetase long-chain family member
CE: Cholesterylester
CRC: Colorectal cancer
ELOVL: Elongation of very long-chain fatty acids protein
FATP: Fatty acid transport protein
FFA: Free fatty acid
IS: Internal standard
LC: Liquid chromatography
MS: Mass spectrometry
PC: Phosphatidylcholine
PE: Phosphatidylethanolamine
PG: Phosphatidylglycerol
PI: Phosphatidylinositol
PL: Phospholipid
PS: Phosphatidylserine
SM: Sphingomyelin
TAG: Triacylglycerol
VLCFA: Very long-chain fatty acid
