# Supplementary material for: Very long-chain fatty acids are accumulated in triacylglycerol and nonesterified forms in colorectal cancer tissues
Source: Sci Rep. 2021 Mar 17;11:6163. doi: 10.1038/s41598-021-85603-w (PMC7969925; doi:10.1038/s41598-021-85603-w)
Supplement: Supplementary file 1 — Supplementary information. [file 41598_2021_85603_MOESM1_ESM.pdf]

Supplemental materials, tables and figures

Very long-chain fatty acids are accumulated in triacylglycerol and nonesterified forms in colorectal cancer tissues

Kotaro Hama[1,*], Yuko Fujiwara[1], Tamuro Hayama[2], Tsuyoshi Ozawa[2], Keijiro Nozawa[2], Keiji Matsuda[2] Yojiro Hashiguchi[2], and Kazuaki Yokoyama[1]

Method validation

Methods for FFA species were validated according to a previous report [1]. A 400 μmol/L stock solution of FFA D_{31}-16:0 as a standard compound was prepared and diluted in methanol to obtain serial dilutions. A 400 μmol/L stock solution of FFA D_4-16:0 as an internal standards (IS) were also prepared in methanol. Twenty microliter of the IS and 40 or 80 μL of each diluted standard compound solution were placed into screw-cap glass tubes. After 2 mL of methanol were added into 50 μL of human EDTA plasma, 1 mL of chloroform (Fujifilm Wako Pure Chemical Corporation) and 0.75 mL of water were added, and the total lipid fraction was extracted by the Bligh & Dyer method. The resulting lower organic phase was evaporated with the EZ-2 centrifugal evaporator, and the resulting precipitate was reconstituted with 0.2 mL of ethanol followed by filtration with a YMC Duo-Filter. For validation of the method, three samples containing 8.0, 80, and 800 pmol of standard per injection were analyzed for quality control (QC) purposes. To generate a linear regression curve, $1/x^2$ was used as a weighting factor. Accuracy was calculated as: [(observed concentration − endogenous concentration)/nominal concentration − 1] × 100 (%) and the coefficient of variation was evaluated to determine measurement precision.

Supplementary Table S1 Calibration curve for FFA species

compound	range (pmol)	weight	linearity slope	Intercept	r^2		precision (CV(%))[a] QC-L[b]	QC-M[b]	QC-H[b]	accuracy (%) QC-L[b]	QC-M[b]	QC-H[b]
FA D_{31}-16:0/ FA D_4-16:0	8-2000	$1/x^2$	0.0099	0.0027	0.984	Intra-day (n = 9)	6.5	3.6	2.8	12.4	2.3	7.2
						Inter-day (n = 3+3+3)	11.5	4.5	6.4	16.0	4.5	13.0

[a]Precision was calculated as the coefficient of variation (CV)

[b]Three samples with 8.0, 80, and 800 pmol of FA D_{31}-16:0 were mixed with 200 pmol of FA D_4-16:0 per injection and were analyzed as quality control compounds QC-L, QC-M, and QC-H, respectively

Supplementary Table S2 Quantification of the acyl-CoA species in the colorectal tissues

Species	Normal (n = 15)		Tumor (n = 15)	
	amount[a]	%	amount[a]	%
14:0-CoA	1.6 ± 0.3	1.6	1.8 ± 0.3	1.7
14:1-CoA	0.7 ± 0.1	0.8	0.8 ± 0.1	0.8
15:0-CoA	0.3 ± 0.1	0.3	0.3 ± 0.1	0.3
16:0-CoA	18.4 ± 4.2	18.8	19.0 ± 3.2	18.3
16:1-CoA	2.4 ± 0.3	2.5	3.2 ± 0.5	3.1
17:0-CoA	1.1 ± 0.3	1.1	1.2 ± 0.2	1.2
17:1-CoA	0.2 ± 0.1	0.2	0.4 ± 0.1	0.4
18:0-CoA	10.5 ± 2.3	10.7	9.6 ± 2.0	9.3
18:1-CoA	26.0 ± 4.5	26.5	30.5 ± 5.8	29.4
18:2-CoA	8.1 ± 1.6	8.3	7.3 ± 0.9	7.0
18:3-CoA	0.5 ± 0.1	0.5	0.4 ± 0.1	0.4
19:0-CoA	0.2 ± 0.0	0.2	0.2 ± 0.0	0.2
19:1-CoA	0.2 ± 0.0	0.2	0.3 ± 0.1	0.3
19:2-CoA	0.1 ± 0.0	0.1	0.1 ± 0.0	0.1
19:4-CoA	0.1 ± 0.0	0.1	0.1 ± 0.0	0.1
20:0-CoA	1.0 ± 0.2	1.1	0.9 ± 0.2	0.8
20:1-CoA	2.4 ± 0.4	2.4	3.8 ± 1.0	3.7
20:2-CoA	1.6 ± 0.3	1.6	2.1 ± 0.4	2.0
20:3-CoA	3.2 ± 0.6	3.2	3.2 ± 0.4	3.1
20:4-CoA	7.0 ± 1.2	7.1	6.6 ± 0.6	6.4
20:5-CoA	1.3 ± 0.3	1.3	0.9 ± 0.1	0.8
20:6-CoA	0.2 ± 0.0	0.2	0.1 ± 0.0	0.1
21:0-CoA	0.1 ± 0.0	0.1	0 ± 0.0	0.0
21:2-CoA	0.1 ± 0.0	0.1	0.1 ± 0.0	0.1
22:0-CoA	0.5 ± 0.1	0.5	0.3 ± 0.1	0.3
22:1-CoA	1.0 ± 0.2	1.0	1.1 ± 0.3	1.0
22:2-CoA	0.4 ± 0.1	0.4	0.5 ± 0.1	0.5
22:3-CoA	0.2 ± 0.0	0.2	0.3 ± 0.1	0.3
22:4-CoA	0.9 ± 0.1	0.9	1.2 ± 0.2	1.2
22:5-CoA	1.3 ± 0.3	1.4	1.5 ± 0.2	1.4
22:6-CoA	2.3 ± 0.5	2.3	2.5 ± 0.2	2.4
23:0-CoA	0.2 ± 0.0	0.2	0.0 ± 0.0*	0.0
23:1-CoA	0.1 ± 0.0	0.1	0.1 ± 0.0	0.1
24:0-CoA	0.6 ± 0.2	0.6	0.2 ± 0.1**	0.2
24:1-CoA	1.1 ± 0.3	1.1	0.8 ± 0.2	0.8
24:2-CoA	0.2 ± 0.0	0.2	0.2 ± 0.0	0.2
24:3-CoA	0.0 ± 0.0	0.0	0.1 ± 0.0	0.1
24:4-CoA	0.2 ± 0.0	0.2	0.2 ± 0.0	0.2
24:5-CoA	0.1 ± 0.0	0.1	0.2 ± 0.1	0.2
24:6-CoA	0.0 ± 0.0	0.1	0.1 ± 0.0	0.1
25:1-CoA	0.2 ± 0.1	0.2	0.1 ± 0.0	0.1
26:0-CoA	0.1 ± 0.0	0.1	0.0 ± 0.0	0.0
26:1-CoA	0.4 ± 0.1	0.4	0.4 ± 0.1	0.4

26:2-CoA	0.1 ± 0.0	0.1	0.1 ± 0.0	0.1
26:5-CoA	0.0 ± 0.0	0.0	0.1 ± 0.0	0.1
26:6-CoA	0.0 ± 0.0	0.0	0.1 ± 0.0	0.1
total	97.9	100.0	103.5	100.0

[a]pmol/mg protein ± s.d., n.q. below the quantitation range. The ratio of peak area for each acyl-CoA/D_{31}-16:0-CoA was used to calculate the amount of each acyl-CoA species

$^{*}p = 0.026$, $^{**}p = 0.039$ (two-tailed paired *t*-test between normal and tumor tissues)

Supplementary Table S3 Quantification of the acyl-CoA species in HEK293T cells

Species	Mock (n = 3)		*ELOVL1* (n = 3)	
	amount[a]	%	amount[a]	%
14:0-CoA	14.5 ± 2.1	5.9	14.9 ± 2.2	4.7
14:1-CoA	1.2 ± 0.1	0.5	0.5 ± 0.9	0.2
15:0-CoA	0.0 ± 0.0	0.0	0.7 ± 1.2	0.2
16:0-CoA	76.0 ± 12.1	31.1	82.8 ± 1.4	26.2
16:1-CoA	14.1 ± 2	5.8	15.1 ± 1.4	4.8
17:0-CoA	3.2 ± 0.2	1.3	4.5 ± 0.2*	1.4
17:1-CoA	0.5 ± 0.8	0.2	1.5 ± 0.2	0.5
18:0-CoA	14.9 ± 0.8	6.1	18.6 ± 1.0**	5.9
18:1-CoA	84.5 ± 11.6	34.6	110.5 ± 9.8	35
18:2-CoA	3.0 ± 0.9	1.2	3.3 ± 1.4	1.0
20:0-CoA	1.2 ± 0.2	0.5	1.5 ± 0.3	0.5
20:1-CoA	7.6 ± 0.6	3.1	7.5 ± 0.1	2.4
20:2-CoA	2.0 ± 0.7	0.8	2.8 ± 0.1	0.9
20:3-CoA	4.4 ± 0.5	1.8	4.3 ± 0.3	1.4
20:4-CoA	6.6 ± 1.5	2.7	6.1 ± 1.2	1.9
22:0-CoA	0.5 ± 0.8	0.2	1.4 ± 0.1	0.4
22:1-CoA	0.0 ± 0.0	0.0	1.2 ± 1.0	0.4
22:5-CoA	2.3 ± 0.6	0.9	2.7 ± 0.5	0.9
22:6-CoA	3.8 ± 0.6	1.6	4.3 ± 0.9	1.4
24:0-CoA	1.0 ± 0.9	0.4	5.9 ± 1.6***	1.9
24:1-CoA	0.7 ± 0.6	0.3	5.2 ± 0.8#	1.6
26:0-CoA	0.0 ± 0.0	0.0	1.2 ± 1.1	0.4
26:1-CoA	1.2 ± 1.1	0.5	14.7 ± 1.1##	4.7
28:1-CoA	1.1 ± 0.0	0.5	4.7 ± 0.7##	1.5
total	244.3	100.0	315.9	100.0

[a]pmol/mg protein ± s.d., n.q. below the quantitation range. The ratio of peak area for each acyl-CoA/D_{31}-16:0-CoA was used to calculate the amount of each acyl-CoA species

$^{*}p = 0.002$, $^{**}p = 0.008$, $^{***}p = 0.002$, $^{\#}p = 0.009$, $^{\#\#}p < 0.001$ (Student's two-tailed t-test between mock and *ELOVL1* transfected HEK293T cells)

Supplementary Table S4 MRM transition for PL and FFA

Lipid species	Q1 (*m/z*)	Q3 (*m/z*)	Collision Energy
PC 15:0/D_7-18:1	753.5	184.0	34
PC 32:0	734.5	184.0	34
PC 34:1	760.5	184.0	34
PC 38:4	810.5	184.0	34
PC 40:0	846.5	184.0	34
PC 42:0	874.5	184.0	34
PC 42:1	872.5	184.0	34
PC 44:1	900.5	184.0	34
PE 15:0/D_7-18:1	711.5	570.5	22
PE 32:0	692.5	551.5	22
PE 34:1	718.5	577.5	22
PE 38:4	768.5	627.5	22
PE 40:0	804.5	663.5	22
PE 42:0	832.5	691.5	22
PE 42:1	830.5	689.5	22
PE 44:1	858.5	717.5	22
PS 15:0/D_7-18:1	755.5	570.5	22
PS 32:0	736.5	551.5	22
PS 34:1	762.5	577.5	22
PS 38:4	812.5	627.5	22
PS 40:0	848.5	663.5	22

PS 42:0	876.5	691.5	22
PS 42:1	874.5	689.5	22
PS 44:1	902.5	717.5	22
PI 15:0/D_7-18:1	847.5	570.5	22
PI 32:0	828.5	551.5	22
PI 34:1	854.5	577.5	22
PI 38:4	904.5	627.5	22
PI 40:0	940.5	663.5	22
PI 42:0	968.5	691.5	22
PI 42:1	966.5	689.5	22
PI 44:1	994.5	717.5	22
PG 15:0/D_7-18:1	759.5	570.5	22
PG 32:0	740.5	551.5	22
PG 34:1	766.5	577.5	22
PG 38:4	816.5	627.5	22
PG 40:0	852.5	663.5	22
PG 42:0	880.5	691.5	22
PG 42:1	878.5	689.5	22
PG 44:1	906.5	717.5	22
SM d18:1/D_9-18:1	738.5	184.0	34
SM 34:1	703.5	184.0	34
SM 36:2	729.5	184.0	34
SM 42:1	815.5	184.0	34

SM 44:1	843.5	184.0	34
FFA D_4-16:0	259.5	259.5	15
FFA D_{31}-16:0	286.5	286.5	15
FFA 16:0	255.5	255.5	15
FFA 18:0	283.5	283.5	15
FFA 18:1	281.5	281.5	15
FFA 18:2	279.5	279.5	15
FFA 20:0	311.5	311.5	15
FFA 20:1	309.5	309.5	15
FFA 20:4	303.5	303.5	15
FFA 20:5	301.5	301.5	15
FFA 22:0	339.5	339.5	15
FFA 22:1	337.5	337.5	15
FFA 22:6	327.5	327.5	15
FFA 24:0	367.5	367.5	15
FFA 24:1	365.5	365.5	15
FFA 26:0	395.5	395.5	15
FFA 26:1	393.5	393.5	15

Supplementary Table S5 Precursor and Product ions for TAG and CE

Lipid species	Precursor ion (*m/z*)	Product ion (*m/z*)
TAG 16:0-16:0-16:0<br>TAG 18:0-16:0-14:0	824.77017	551.50339
TAG 18:1-16:0-16:0<br>TAG 18:0-16:0-16:1	850.78582	577.51904
TAG 18:1-18:1-16:0<br>TAG 18:0-18:1-16:1	876.80147	603.53469
TAG 24:0-16:0-16:0<br>TAG 18:0-18:0-14:0	936.89537	551.50339
TAG 24:0-18:1-16:0<br>TAG 24:0-18:0-16:1<br>TAG 24:0-20:1-14:0	962.91102	577.51904
TAG 24:0-18:1-18:1<br>TAG 24:0-18:0-18:2	988.92667	603.53469
TAG 26:0-16:0-16:0	964.92667	551.50339
TAG 26:0-18:1-16:0<br>TAG 26:0-18:0-16:1	990.94232	577.51904
TAG 26:0-18:1-18:1<br>TAG 26:0-18:0-18:2	1016.95797	603.53469
CE 16:0	642.61836	369.35158
CE 18:0	670.64966	369.35158
CE 18:1	668.63401	369.35158
CE 18:2	666.61836	369.35158
CE 20:0	698.68096	369.35158
CE 20:1	696.66531	369.35158
CE 20:4	690.61836	369.35158
CE 20:5	688.60271	369.35158
CE 22:0	726.71226	369.35158
CE 22:1	724.69661	369.35158
CE 22:6	714.61836	369.35158
CE 24:0	754.74356	369.35158
CE 24:1	752.72791	369.35158
CE 26:0	782.77486	369.35158
CE 26:1	780.75921	369.35158
PC 15:0/D_7-18:1	753.61337	184.07389

Supplementary Table S6 Oligonucleotide sequence used in quantitative real-time RT-PCR analysis

Gene name	sense (5' to 3')	antisense (5' to 3')
ACTB	ATGAAGATCAAGATCATTGCTCCTC	ACATCTGCTGGAAGGTGGACA
ELOVL1	TTATTCTCCGAAAGAAAGACGGG	ATGACATGCACGGAAGAGTTTAT
ELOVL2	ATGTTTGGACCGCGAGATTCT	CCCAGCCATATTGAGAGCAGATA
ELOVL3	CTGTTCCAGCCCTATAACTTCG	GAATGAGGTTGCCCAATACTCC
ELOVL4	AAGGACCGAGAACCTTTTCAGA	TCCCGCATTATATGATCCCATGA
ELOVL5	TAACAGGAGTATGGGAAGGCA	ACCAGAGGACACGGATAATCTT
ELOVL6	AACGAGCAAAGTTTGAACTGAGG	TCGAAGAGCACCGAATATACTGA
ELOVL7	GCCTTCAGTGATCTTACATCGAG	AGGACATGAGGAGCCAATCTT
ABCD1	GGCTGTCCCCATCATCACT	GCTCCTCCTCCTTCTTTTCCA

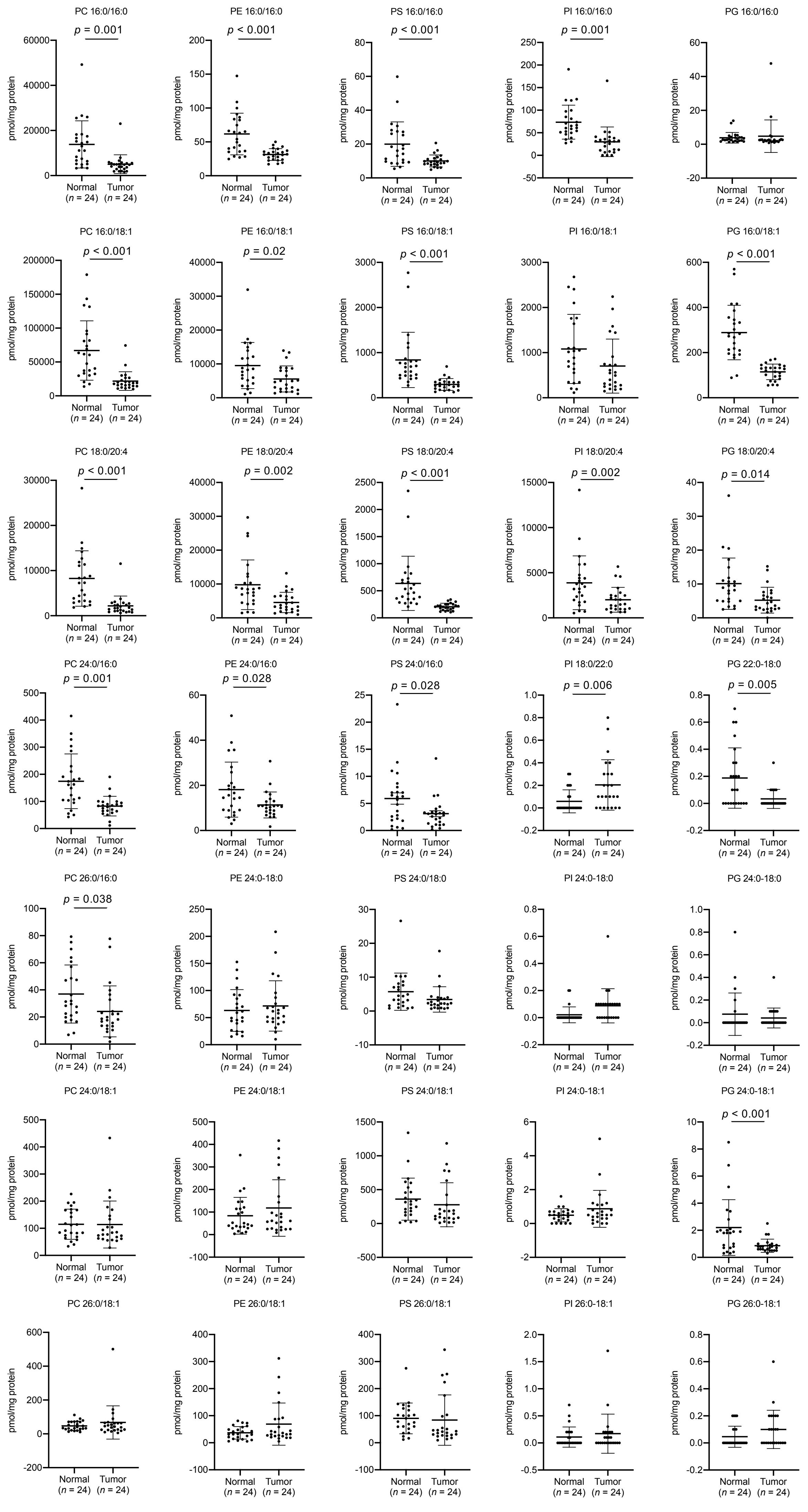

Fig. S1 Quantities of phospholipid (PC, PE, PS, PI and PG) and SM species in colorectal cancer (CRC) tissues

The amount of each phospholipid and SM species was quantified by LC-MS in the positive ion mode. The acyl-moieties for each phospholipid species were determined by LC-MS/MS/MS in the negative ion mode. Data represent the mean ± SD. Statistical analysis was performed using the two-tailed paired *t*-test.

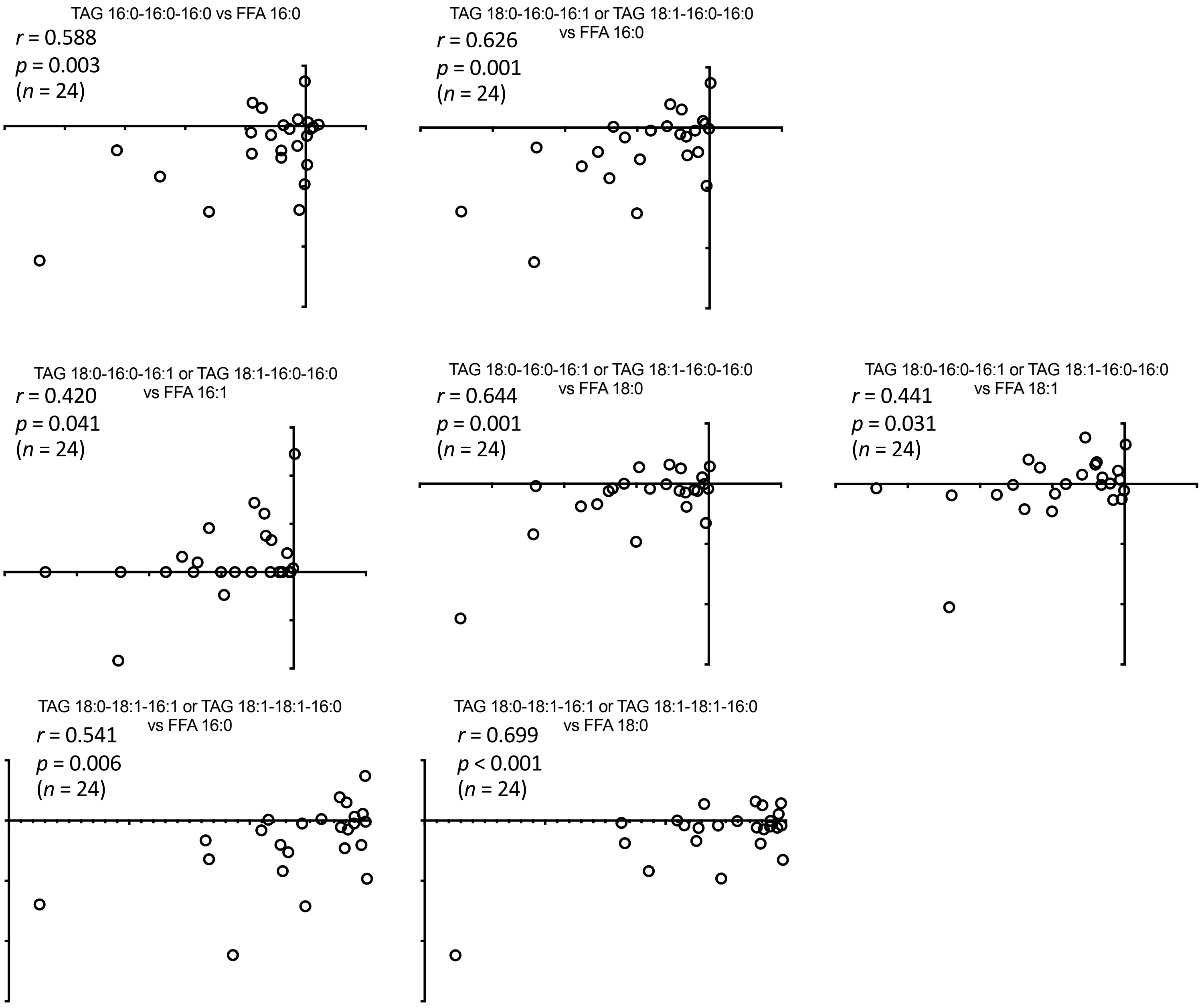

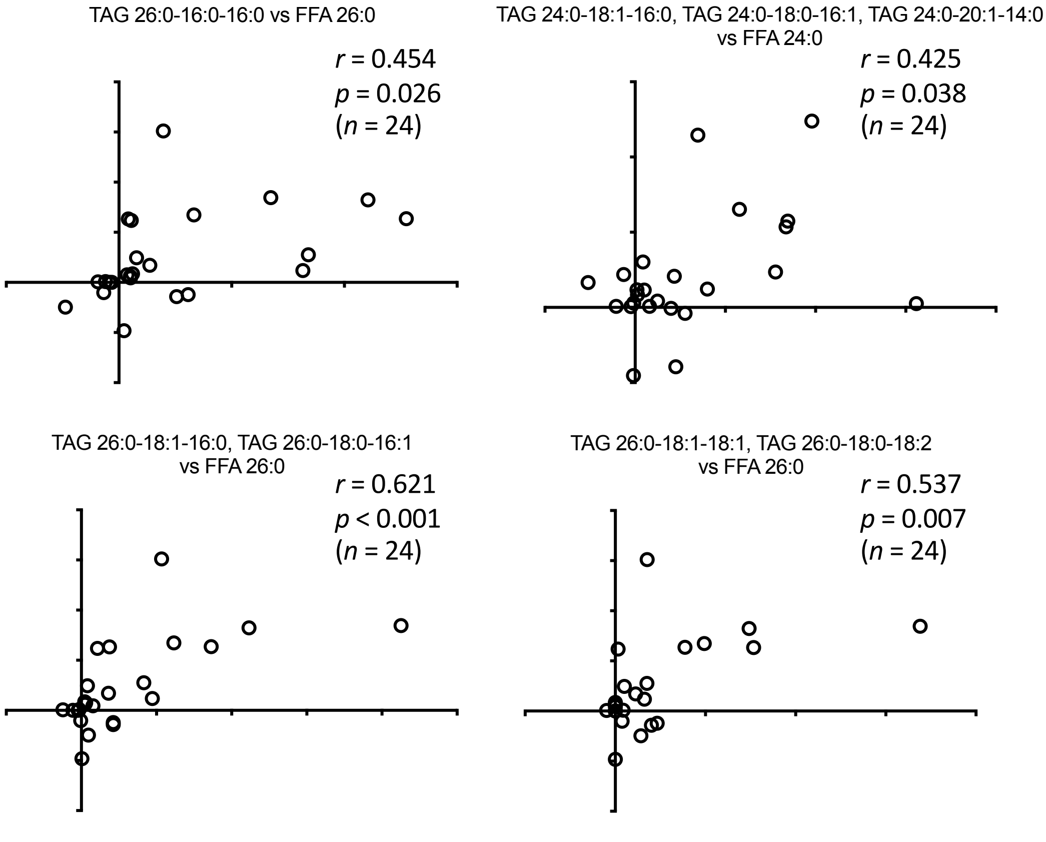

Fig. S2 The correlation between the difference (colorectal cancer − normal) of TAG (x-axis) and FFA (y-axis) species. The values of each significantly correlated ($p < 0.05$) TAG and FFA species are plotted in each panel. Statistical analysis was performed using the Pearson's correlation coefficient.

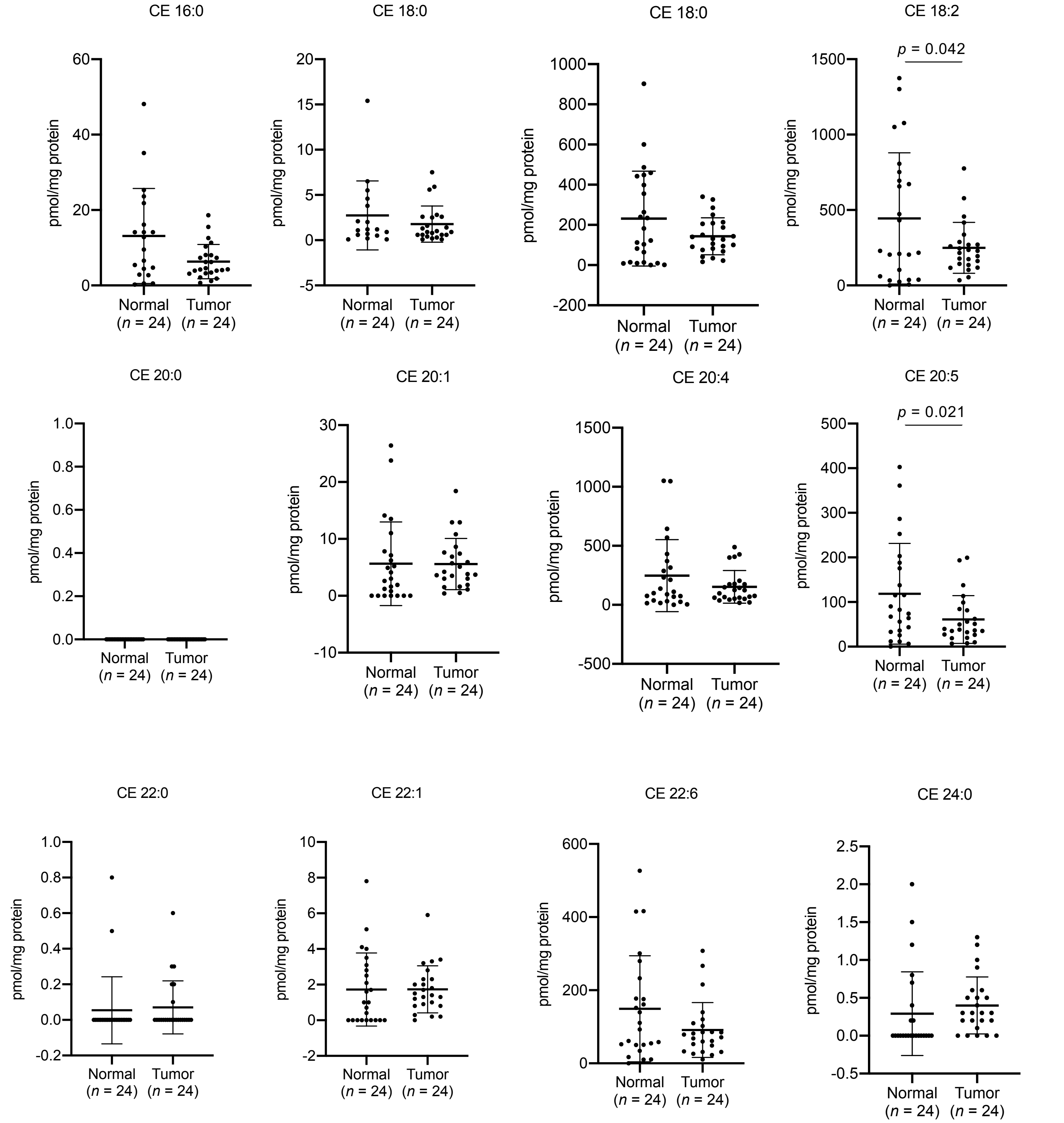

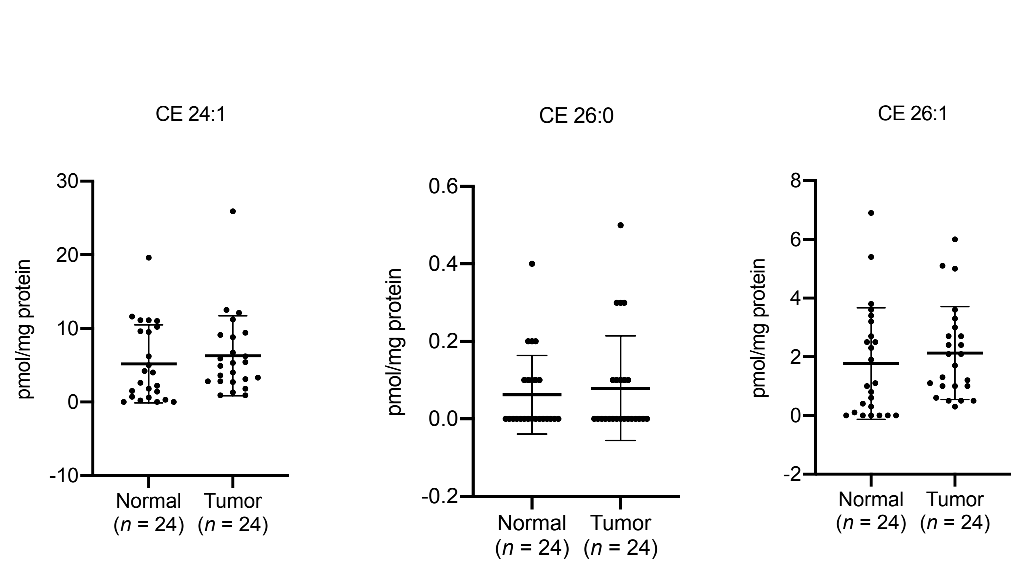

Fig. S3 Quantities of each cholesterylester (CE) species in the colorectal cancer (CRC) tissues

The amount of each CE species was quantified using the parallel reaction monitoring (PRM) mode in the positive ion mode. Data represent the mean ± SD. Statistical analysis was performed using the two-tailed paired *t*-test.

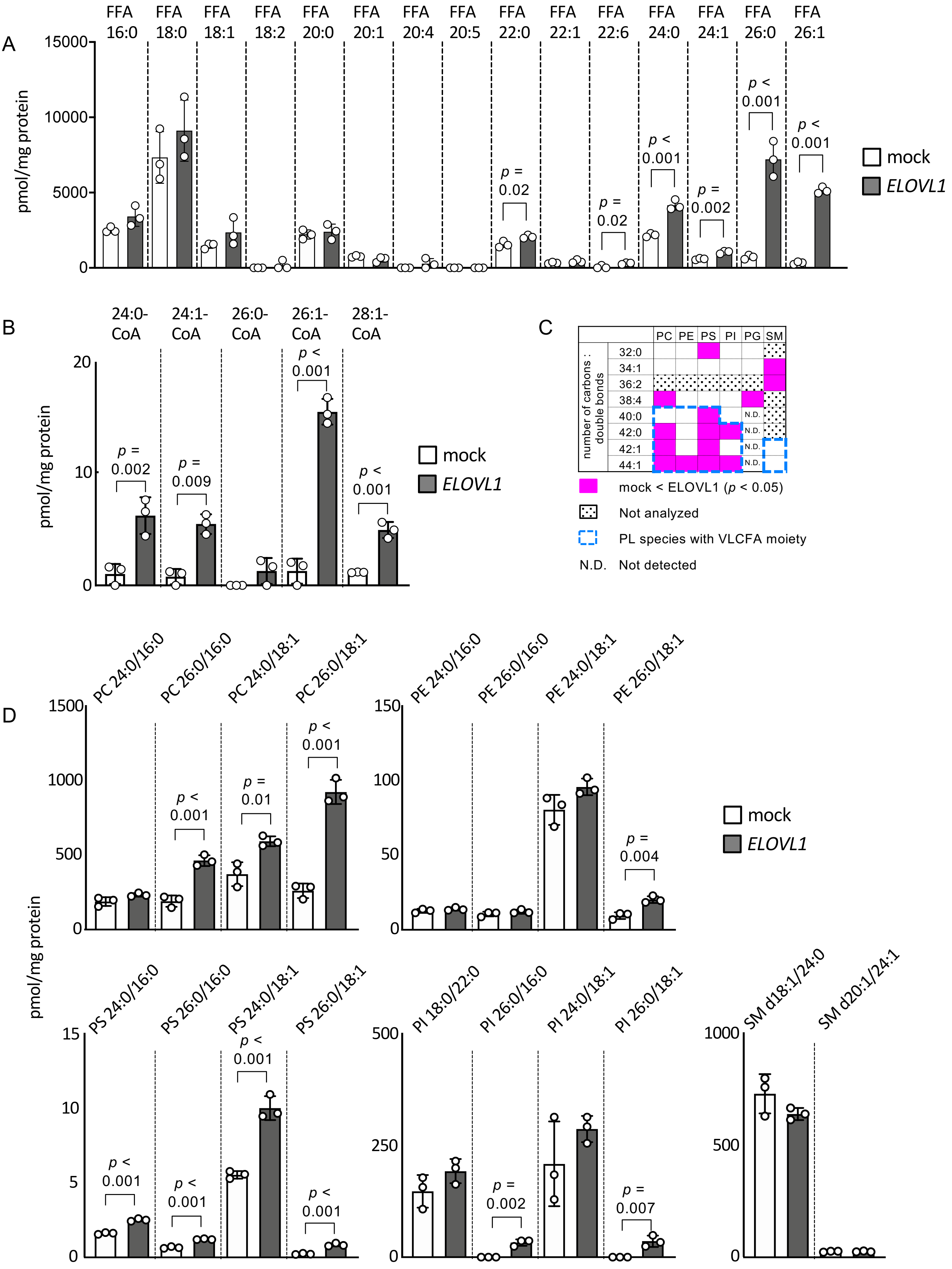

Fig. S4 Continued

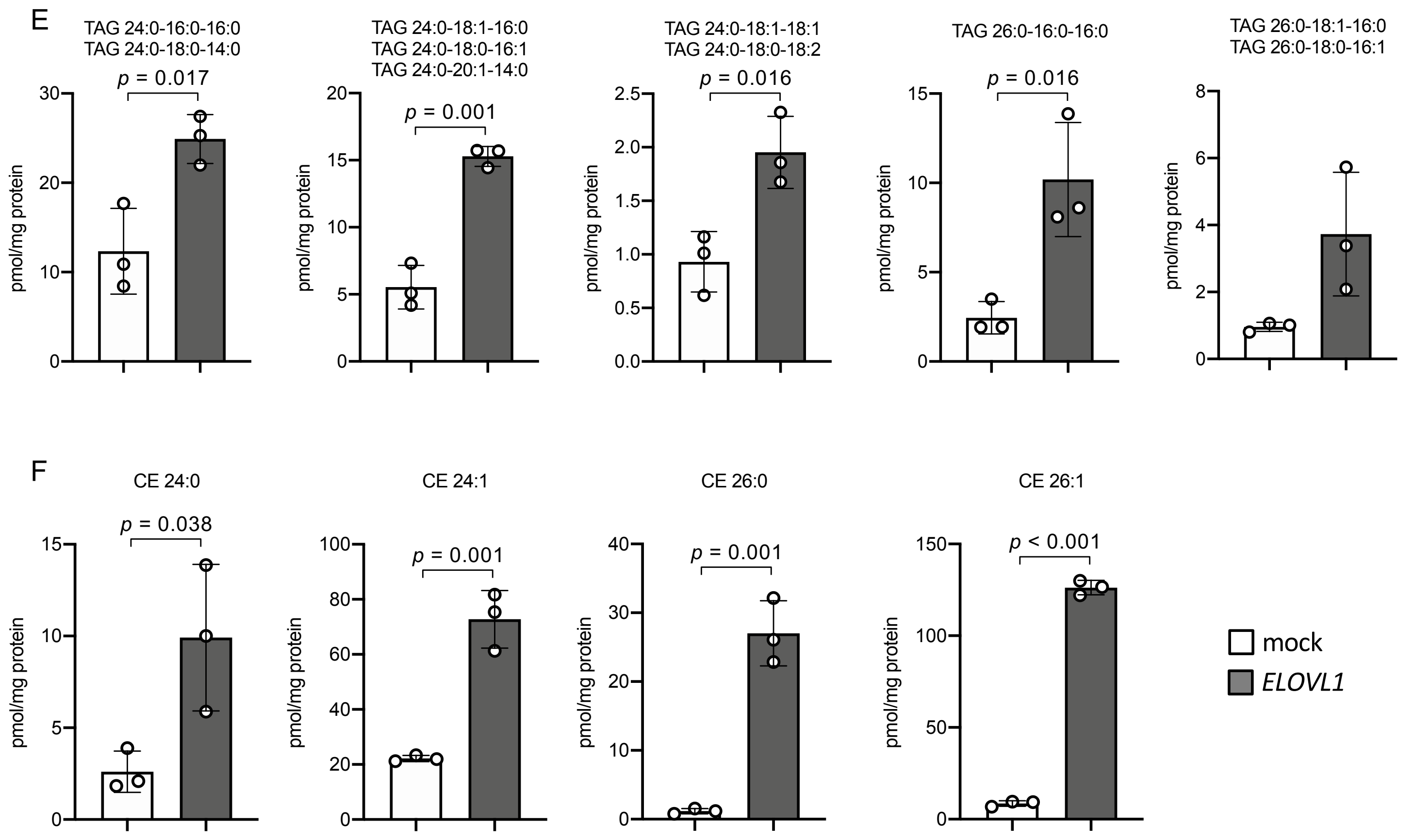

Fig. S4 The amount of FFA , acyl-CoA, PL, TAG and CE species in HEK293T cells transfected with pcDNA3.1-human *ELOVL1*.

(A, B) The amount of each FFA (A) and acyl-CoA (B) species in HEK293T transfected with *ELOVL1*. (C) PL species present in quantities significantly higher in ELOVL1-overexpressing cells than in mock transfected cells are given in magenta. PL species for which the multiple reaction monitoring (MRM) channels were not designed are indicated in the dot box. PL species with a VLCFA moiety (C24:0 and C26:0) are indicated by blue dashed line. (D-F) The amount of each PL (D), TAG (E) and CE (F) species with a VLCFA moiety is represented. Both the nonesterified (FFA) and esterified VLCFA (acyl-CoA, PL, TAG and CE) were significantly accumulated in HEK293T cells transfected with *ELOVL1*. Data represent the mean ± SD. Statistical analysis was performed using the Student's two-tailed *t*-test in (A-F).

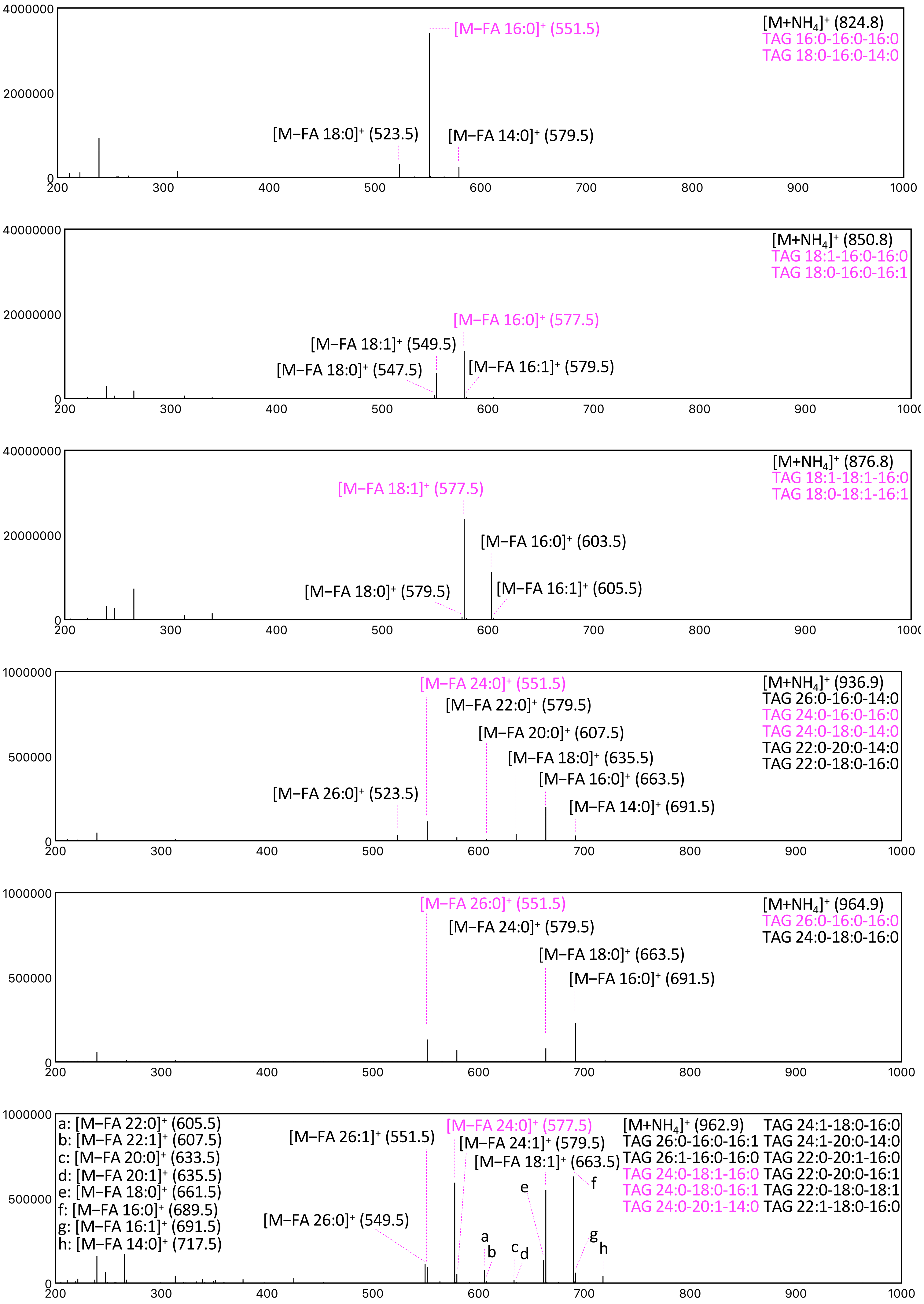

Fig. S5 Continued.

X-axis: m/z, Y-axis: spectral intensity (cps)

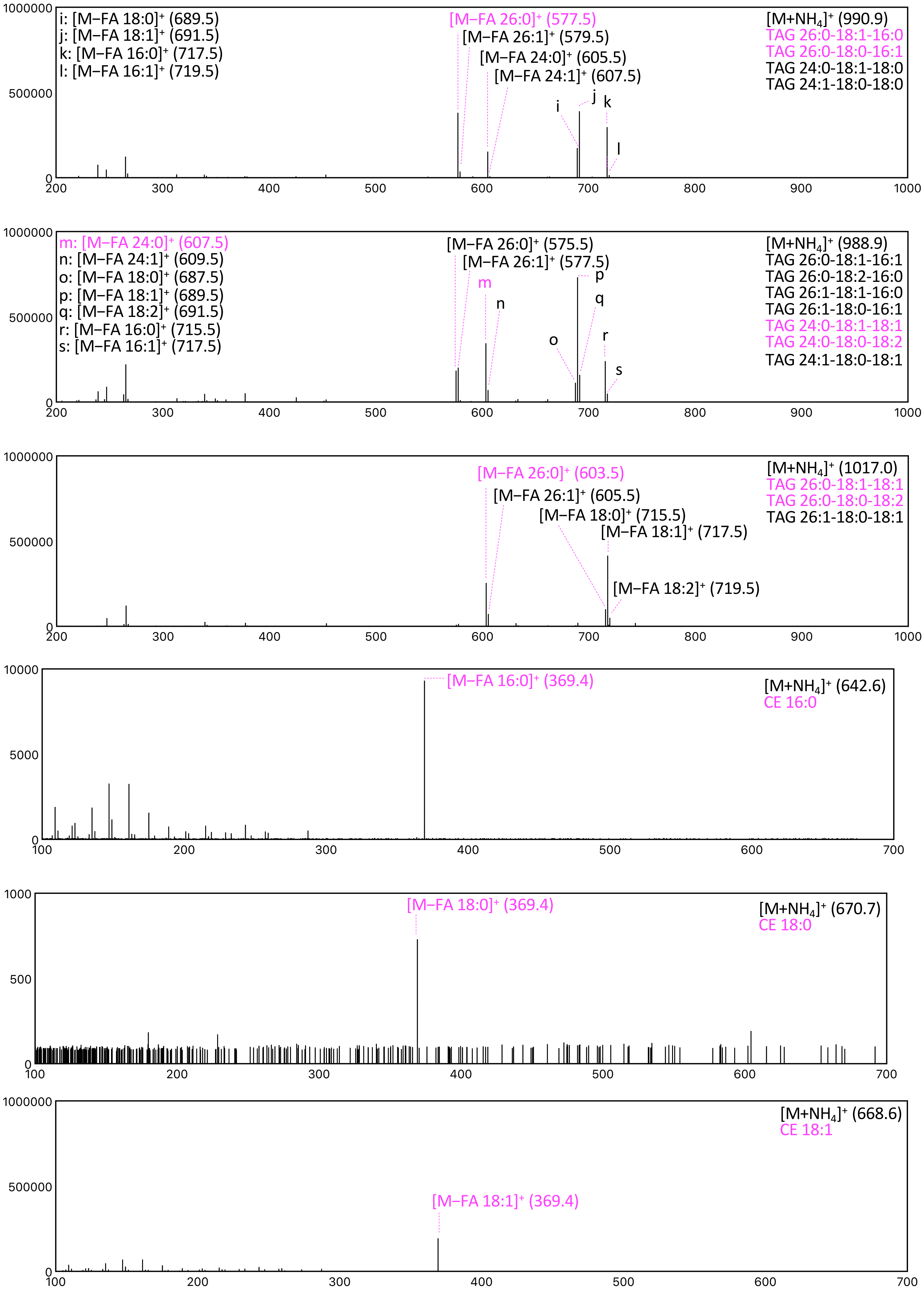

Fig. S5 Continued.

X-axis: m/z, Y-axis: spectral intensity (cps)

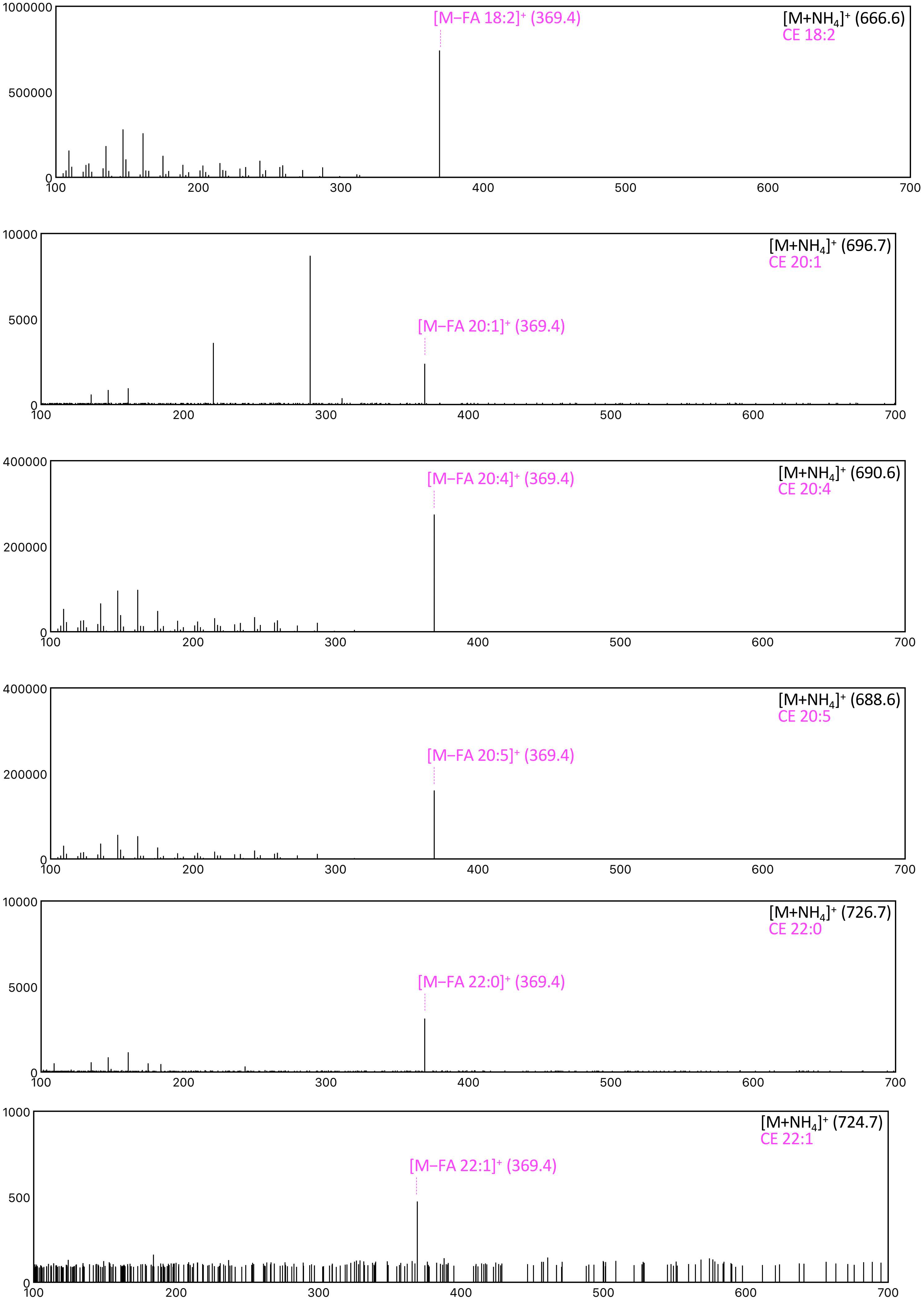

Fig. S5 Continued.

X-axis: m/z, Y-axis: spectral intensity (cps)

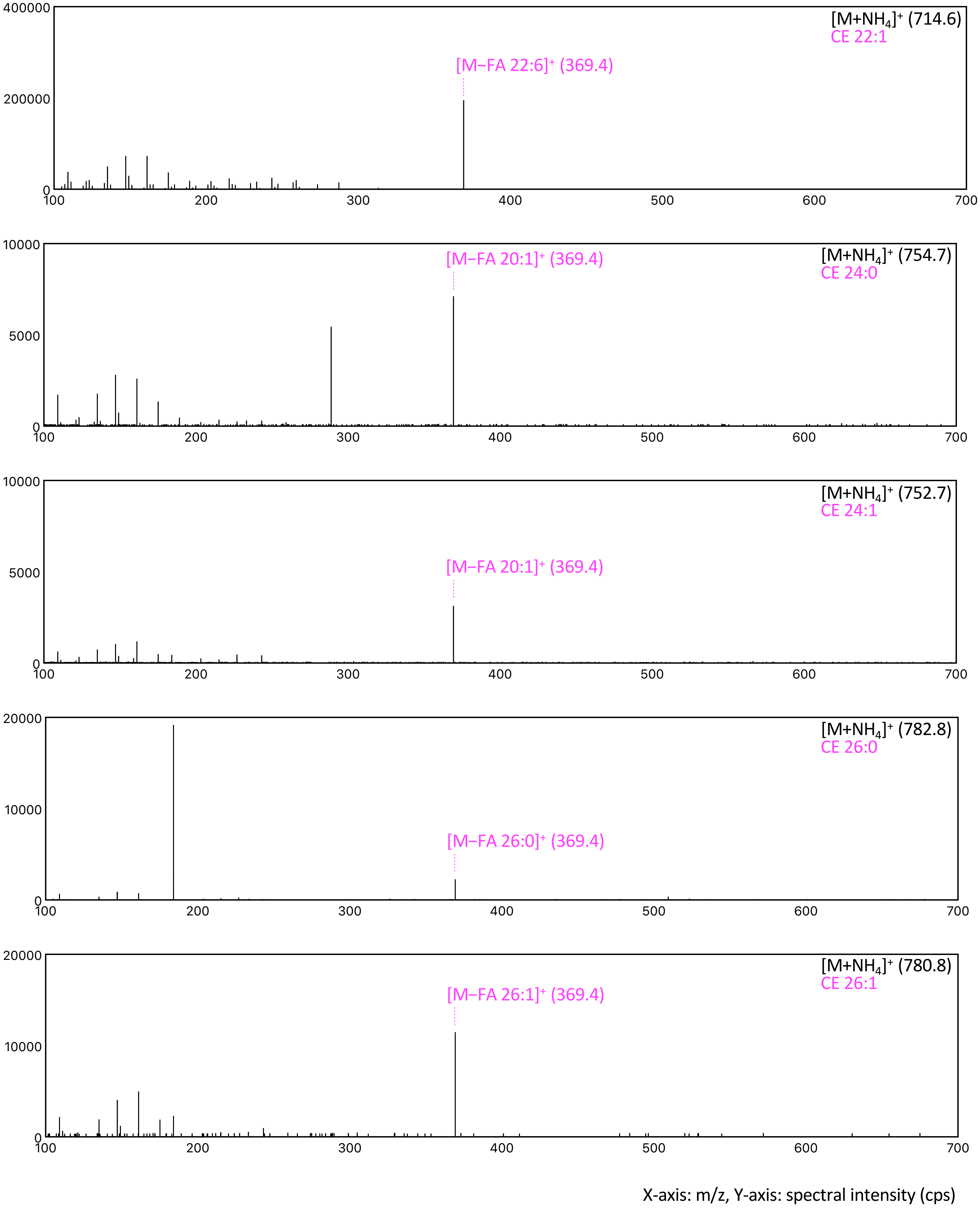

Fig. S5 The product ion spectra of specific *m/z* signals of TAG or CE species in CRC tissues. All spectral data were obtained by parallel reaction monitoring (PRM) mode in the positive ion mode. Quantified TAG or CE species and the spectra used to obtain the chromatogram for quantitation analysis are represented in magenta.
